# A two-tier feature selection method for predicting mortality risk in ICU patients with acute kidney injury

**DOI:** 10.1038/s41598-024-63793-3

**Published:** 2024-07-22

**Authors:** Mengqing Liu, Zhiping Fan, Yu Gao, Vivens Mubonanyikuzo, Ruiqian Wu, Wenjin Li, Naiyue Xu, Kun Liu, Liang Zhou

**Affiliations:** 1 College of Health Science and Engineering University of Shanghai for Science and Technology, Shanghai, 200093 China; 2 Shanghai University of Medicine & Health Sciences, Shanghai, 201318 China; 3 Jiading District Central Hospital Affiliated to Shanghai University of Medicine & Health Sciences, Shanghai, 201899 China; 4 Research Center for Medical Intelligent Development, China Hospital Development Institute, Shanghai Jiao Tong University, Shanghai, 200025 China

**Keywords:** Acute kidney injury (AKI), Boruta, XGBoost, Explainable model, Two-tier feature selection, Computational models, Predictive medicine, Machine learning, Kidney diseases

## Abstract

Acute kidney injury (AKI) is one of the most important lethal factors for patients admitted to intensive care units (ICUs), and timely high-risk prognostic assessment and intervention are essential to improving patient prognosis. In this study, a stacking model using the MIMIC-III dataset with a two-tier feature selection approach was developed to predict the risk of in-hospital mortality in ICU patients admitted for AKI. External validation was performed using separate MIMIC-IV and eICU-CRD. The area under the curve (AUC) was calculated using the stacking model, and features were selected using the Boruta and XGBoost feature selection methods. This study compares the performance of a stacking model using two-tier feature selection with a model using single-tier feature selection (XGBoost: 85; Boruta: 83; two-tier: 0.91). The predictive effectiveness of the stacking model was further validated by using different datasets (Validation 1: 0.83; Validation 2: 0.85) and comparing it with a simpler model and traditional clinical scores (SOFA: 0.65; APACH IV: 0.61). In addition, this study combined interpretable techniques and causal inference to analyze the causal relationship between features and predicted outcomes.

## Introduction

Acute kidney injury (AKI), a significant factor to inpatient mortality worldwide, affects approximately one-fifth of hospitalized individuals^1–3^. The International Society of Nephrology's 0 by 25 initiative aims to eradicate preventable AKI-related deaths by the year 2025^4^. Despite considerable recent efforts, identifying effective treatments that substantially enhance renal recovery remains challenging. Early prediction or detection of AKI carries significant clinical implications but poses a substantial hurdle. Early prediction or detection of AKI has significant clinical implications but poses a substantial challenge. To address the limitations of early AKI prediction, researchers have increasingly turned to machine learning methods. However, the success of these models hinges on the selection of relevant features. To this end, diverse feature selection techniques are employed to improve model generalization, stability, and interpretability^5–7^.

In this context, artificial intelligence (AI) has demonstrated promise for time-sensitive applications in AKI. These applications encompass early identification, warning, and the provision of AKI treatment recommendations^8,9^. Machine learning-based models can detect AKI at an early stage, providing clinicians with a chance to intervene earlier and potentially improve patient outcomes^10–12^. While previous research on AKI prediction has predominantly focused on specific settings such as hospital-acquired AKI^13^, postoperative AKI^14^, cancer-related AKI^8^, and critically ill patients in intensive care units (ICUs)^15,16^, as well as patients admitted to emergency departments^17^, there remains a gap in machine learning models for predicting AKI in general and critically ill patients. The high heterogeneity of patient history data in general hospitals presents a significant challenge for the independent validation of predictive models^18^. This is particularly problematic for current machine learning-based mortality risk prediction models, as they often rely on a multitude of patient test results, such as routine blood tests, as input features. The multiplicity of features and the lengthy data collection process pose a significant challenge to model application. Consequently, reducing the number of input features while preserving model accuracy has become a pressing concern. Zhu et al.^19^ successfully developed a machine learning model for predicting the risk of death in sepsis patients, achieving a 71% reduction in the number of features. Their findings indicate that even with small samples and low-dimensional data, accurate identification of patients at risk is feasible, enabling early treatment. Additionally, Shen et al.^20^ and Wu et al.^7^ validated the impact of feature reduction on the stability and accuracy of model prediction performance. Although many models have been proposed to identify patients at risk for AKI, few models can predict the risk of clinically important outcomes (hospital death or dialysis) once a patient develops AKI. The application of models with clinically important predictions may help guide the early treatment of patients with AKI.

In this study, we introduce a machine learning model that employing a two-stage feature selection process to predict in-hospital mortality risk among ICU patients with AKI. Our aim is to identify crucial features for mortality prediction and, as a result, reduce feature dimensionality to enhance model interpretability without sacrificing accuracy.

## Results

### Study population characteristics

For this study, data from 16,090 initial ICU admissions in the MIMIC III database were collected, with 11,182 patients meeting the inclusion criteria. These data comprised the training set, with 30% allocated for internal validation. Patients were categorized as either dead or surviving. Furthermore, data from MIMIC-IV (validation 1) and eICU-CRD (validation 2) were retrieved for external validation using the same criteria. The mortality and survival data for the training set, internal validation set, and external validation set were statistically analyzed a summarized in Table 1. The training set consisted of 7828 cases, of which 2273 resulted in mortality and 5555 in survival. The internal validation set consisted of 3354 cases with 840 deaths and 2514 survivors (see Supplementary Table 1). External validation set 1 (MIMIC-IV) included 7822 cases with 6705 deaths and 1117 survivors. External validation set 2 (eICU-CRD) consisted of 5928 cases, with 5403 survivors and 525 deaths. Across all three datasets, the proportion of men diagnosed with AKI exceeded that of women, and correspondingly, the mortality rate was also higher among male patients. Additionally, AKI patients aged over 60 exhibited a notably elevated mortality rate compared to patients in other age brackets.

**Table 1:** The baseline characteristics of AKI patients are analyzed.

Characteristics	Train DataSet 7828		Validation 1 7822		Validation 2 5928	
	Survival 5555	Death 2273	Survival 6705	Death 840	Survival 5403	Death 525
Age (%)						
18 < age < 30	198 (3.56)	27 (1.18)	267 (3.98)	4 (0.47)	228 (4.21)	10 (1.90)
1030 < age < 60	1880 (33.84)	517 (22.74)	3075 (48.86)	193 (22.98)	1768 (32.72)	141 (26.85)
> 60	3477 (62.59)	1729 (76.07)	3363 (50.15)	643 (76.55)	3395 (62.83)	373 (71.04)
Gender (%)						
Female	2411 (43.4)	1038 (45.7)	2827 (42.2)	359 (42.7)	2096 (38.8)	217 (41.3)
Male	3144 (56.6)	1235 (54.3)	3878 (57.8)	481 (57.3)	3307 (61.2)	308 (58.7)
BUN (mmol/L)	24.09 (16.04)	22.95 (14.89)	24.74 (16.48)	33.63 (18.70)	24.53 (20.71)	34.87 (24.28)
CL (mmol/L)	105.54 (5.32)	105.78 (5.19)	100.10 (3.71)	105.01 (6.17)	105.44 (6.95)	103.92 (7.65)
Creatinine(mg/dL)	1.32 (1.07)	1.27 (1.03)	1.41 (1.33)	1.78 (1.30)	1.53 (1.68)	2.12 (1.65)
FiO_2_ (%)	60.56 (22.93)	60.12 (23.09)	60.56 (22.93)	60.12 (23.09)	29.52 (36.45)	43.62 (40.99)
GCS	4.07 (1.05)	4.03 (1.05)	14.79 (1.04)	3.42 (1.43)	15.23 (1.25)	6.25 (1.42)
GLU (mmol/L)	133.25 (40.32)	132.41 (37.54)	100.10 (3.71)	142.70 (47.24)	178.94 (129.09)	210.12 (141.86)
HCO_3_ (mmol/L)	18.05 (8.45)	18.29 (8.30)	18.05 (8.45)	18.29 (8.30)	21.69 (3.14)	19.78 (3.66)
HCT (%)	31.92 (5.35)	31.96 (5.31)	34.15 (6.22)	31.69 (5.72)	33.15 (5.22)	35.12 (6.01)
HR (bpm)	84.80 (15.05)	84.27 (14.67)	85.10 (15.23)	88.44 (17.17)	101.05 (29.22)	110.00 (33.32)
HB (g/L)	10.79 (1.81)	10.85 (1.79)	11.51 (3.21)	10.51 (1.85)	10.81 (3.01)	11.51 (2.25)
INR	1.34 (0.53)	1.31 (0.46)	1.32 (0.62)	1.56 (0.71)	1.39 (0.54)	1.70 (1.05)
K (mmol/L)	4.19 (0.99)	4.24 (3.28)	5.27 (2.03)	4.26 (0.65)	4.16 (0.71)	4.36 (0.97)
Na (mmol/L)	138.34 (3.68)	138.25 (3.67)	136.50 (14.18)	138.35 (4.79)	137.86 (7.50)	138.45 (7.60)
PCO_2_ (mmHg)	47.80 (10.36)	48.34 (11.46)	47.20 (10.30)	45.77 (11.73)	46.10 (9.30)	43.67 (11.43)
PEEP (cmH_2_O)	5.90 (1.15)	5.87 (1.17)	5.95 (1.23)	6.41 (1.53)	5.40 (0.49)	5.64 (0.48)
PH	6.71 (0.77)	6.71 (0.78)	6.76 (0.75)	6.75 (0.72)	3.01 (4.18)	4.11 (4.06)
PLT (10^9^/L])	207.53 (88.25)	207.73 (84.94)	248.35 (138.17)	194.53 (105.55)	105.02 (274.71)	232.92 (567.33)
PO_2_ (mmHg)	140.56 (61.22)	144.63 (62.31)	143.14 (62.51)	126.46 (55.62)	69.31 (89.80)	54.16 (72.74)
PT (s)	15.62 (45.17)	15.27 (24.16)	14.56 (6.58)	17.63 (9.67)	15.85 (5.26)	15.85 (5.26)
PTT (s)	35.70 (12.22)	35.41 (12.28)	36.17 (12.57)	40.22 (14.91)	37.70 (14.21)	43.87 (19.72)
Pain	2.11 (2.13)	2.12 (2.18)	2.13 (2.16)	1.27 (1.84)	2.22 (1.86)	1.17 (2.24)
Resp (bpm)	18.00 (3.97)	18.04 (3.96)	11.53 (9.55)	19.53 (4.60)	23.04 (15.21)	28.24 (14.54)
SpO_2_ (%)	96.96 (5.00)	97.13 (9.00)	87.43 (15.43)	96.29 (3.86)	84.03 (14.53)	92.89 (5.36)
Temp (°C)	36.77 (0.54)	36.76 (0.53)	37.38 (1.14)	36.63 (0.70)	34.83 (7.33)	36.49 (6.52)
Tidal volume (mL)	491.96 (75.82)	491.83 (74.88)	475.52 (82.10)	484.68 (77.49)	457.32 (72.10)	488.68 (78.46)
WBC (cells/μL)	11.45 (5.09)	11.53 (5.04)	11.63 (5.43)	12.53 (6.81)	13.27 (8.72)	15.66 (13.12)

Statistical analysis of mortality and survival data within the training, internal validation, and external validation sets is conducted. Age and gender are depicted as frequencies and proportions within various categories, whereas other indicators are expressed using their mean values and standard deviations.

### Feature selection and Model performance

Feature selection involved initial screening with the Boruta algorithm for the first tier (see Supplementary Fig. 1A,B). Subsequently refinement was carried out using XGBoost for the second tier (see Supplementary Fig. 2A). Ultimately, a total of 24 relevant features were identified. Additionally, the features identified using only XGBoost feature selection are depicted in Supplementary Fig. 2B.

**Figure 1:**
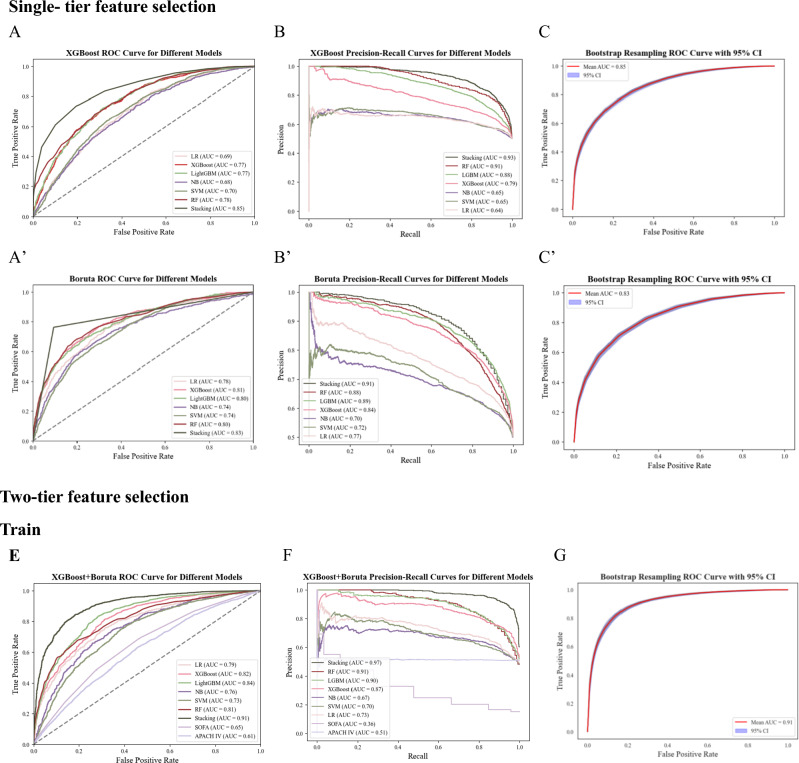

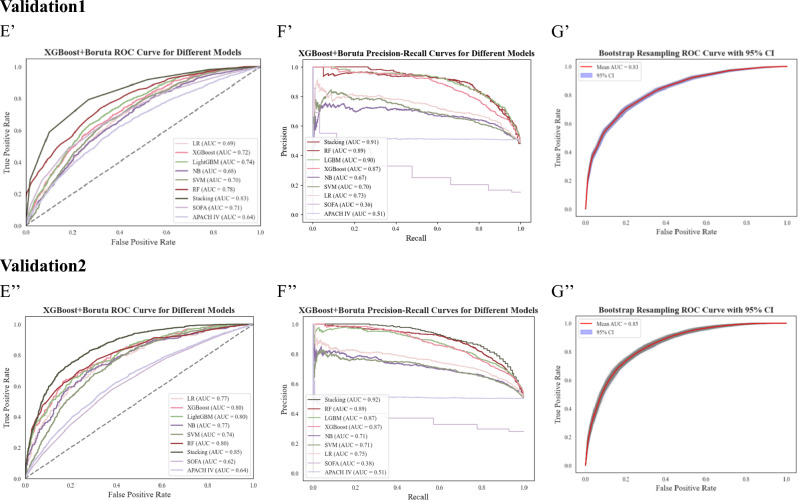
Illustrates model performance metrics with single- and two-tier feature selection, including feature selection using only XGBoost and Boruta, along with receiver operating characteristic curves (ROC), precision-recall curves (PRC) predicted by models with two-tier feature selection. These evaluations are conducted on both the training set (MIMIC-III) and the validation sets (MIMIC-IV, eICU-CRD), with the receiver operating characteristic curves presented alongside a shaded 95% confidence interval (ROC (95% CI)). (**A**–**C**) denote the ROC, PRC, and ROC (95% CI) predicted by the model using only XGBoost for feature selection, respectively. (**A′**–**C′**) denote the ROC, PRC, and ROC (95% CI) predicted by the model using only Boruta for feature selection. (**E**–**G**) denote the ROC, PRC, and ROC (95% CI) predicted by the model using two-tier feature selection on the training set. (**E′**–**G′**) denotes the AUROC, PRC, and ROC (95% CI) predicted by the model using two-tier feature selection on Validation 1 (MIMIC-IV). (**E″**–**G″**) denotes AUROC, PRC, and ROC (95% CI) predicted by the model using two-tier feature selection on Validation2 (eICU-CRD)**.** *CI* confidence interval.

**Figure 2:**
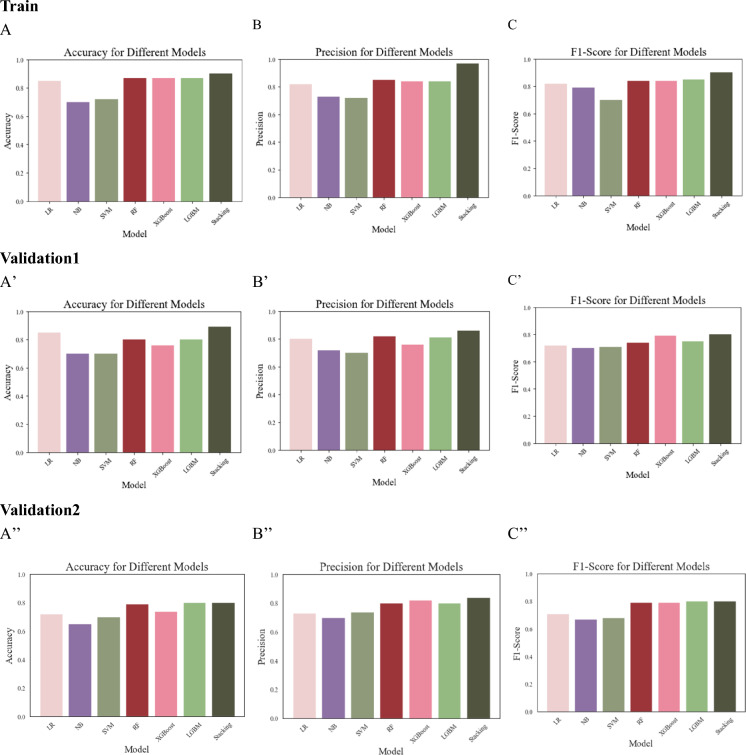
Comparison of training and validation set precision, accuracy, and f1 scores for the best performing model (**A**,**A′**,**A**″): Accuracy; (**B**,**B′**,**B″**): Precision; (**C**,**C′**,**C″**): F1-Score.

The construction of the model is carried out according to the features screened by the Boruta and XGBoost algorithms, respectively, and the prediction effect of the model is evaluated and compared. The evaluation indexes are shown in Fig. 1A–C,A–C′′). As can be seen from the figure, the best-evaluated algorithm is stacking, with an AUC (95% CI) of 0.85 (0.846–0.854) for XGBoost-Stacking (Stacking model construction using XGBoost filtered features) and an AUC (95% CI) of 0.83 (0.828–0.831) for Boruta-Stacking (Stacking model construction using Boruta filtered features). Subsequently, model construction was performed using two-tier feature selection, and different types of algorithms were used for model training. The results showed that the stacking algorithm gave the best prediction with an AUC (95% CI) of 0.91 (0.906–0.915) (Fig. 1E–G). The precision, accuracy, and F1-score of the trained model were evaluated, achieving values of 0.90, 0.89, and 0.90, respectively (Fig. 2A–C). Furthermore, a comparison was conducted between the performance of models developed using single-tier and two-tier feature selection methods (Table 2; see also Supplementary Table 2 for detailed results). This analysis revealed that the two-tier feature selection approach consistently yielded superior prediction performance compared to the single-tier method.

**Table 2:** Evaluation of models for predicting AKI with two-tier feature selection.

Models	AUC	Accuracy	Precision	Recall	F1 score
Support vector machine	0.73	0.72	0.72	0.70	0.70
Naïve Bayes	0.76	0.70	0.73	0.72	0.70
Logistic regression	0.79	0.86	0.82	0.86	0.82
Random forest	0.81	0.87	0.85	0.87	0.84
Extreme gradient boosting	0.82	0.87	0.84	0.87	0.84
Light gradient boosting machine	0.84	0.87	0.84	0.84	0.85
Stacking	0.91	0.90	0.89	0.93	0.90

*AUC* area under the curve.

To further validate the predictive effectiveness of the developed two-tier feature selection model, we used both internal and external validation sets to evaluate the model's performance. Figure 1E’–G’ demonstrates the validation results on validation set 1, with an AUC (95% CI) of 0.83 (0.830–0.833). Figure 2A’–C’ shows the precision of 0.86, accuracy of 0.81, and F1-score of 0.80 for validation set 1. In Fig. 1E”–G”, we show the validation results of validation set 2 with an AUC (95% CI) of 0.85 (0.845–0.853). Figure 2 A”–C” shows that validation set 2 has a precision of 0.84, an accuracy of 0.80, and an F1-score of 0.80. Comparing the training results with the validation results reveals (Tables 2 and 3) that the AUC values of both the internal and external validation sets are higher than 0.80, indicating that the model predicts well on different datasets. In addition, we compared the constructed model with the traditional clinical scoring systems SOFA and APACHE IV (Fig. 1E, E’, E”). The results showed that on the training set, the AUC was 0.65 for SOFA and 0.61 for APACHE; on the validation set 1, the AUC was 0.71 for SOFA and 0.64 for APACHE; and on the validation set 2, the AUC was 0.62 for SOFA and 0.64 for APACHE. These results indicate that, compared to the traditional clinical scoring system, the constructed model has a better prediction effect. The experimental results of internal validation are presented in Online Supplementary Fig. 3A–C, Fig. 4A–C, and Table 3.

**Table 3:** Model performance metrics.

Models	Validation 1					Validation 2				
	AUC	Accuracy	Precision	Recall	F1 score	AUC	Accuracy	Precision	Recall	F1 score
SVM	0.70	0.71	0.69	0.70	0.71	074	0.68	0.70	0.65	0.66
NB	0.68	0.71	0.70	0.73	0.71	0.77	0.70	0.65	0.63	0.67
LR	0.69	0.73	0.71	0.72	0.72	0.77	0.73	0.72	0.73	0.71
RF	0.79	0.77	0.76	0.75	0.74	0.80	0.80	0.79	0.77	0.79
XGBoost	0.80	0.71	0.75	0.72	0.79	0.80	0.82	0.74	0.76	0.79
LGBM	0.82	0.80	0.79	0.73	0.79	0.80	0.84	0.80	0.78	0.80
Stacking	0.83	0.86	0.81	0.79	0.80	0.85	0.84	0.80	0.79	0.80

Internal and external validation of two-tier feature selection. *SVM* Support vector machine, *NB* Naïve Bayes, *LR* logistic regression, *RF* random forest, *XGBoost* extreme gradient boosting, *LGBM* light gradient boosting machine, *AUC* area under the curve.

**Figure 3:**
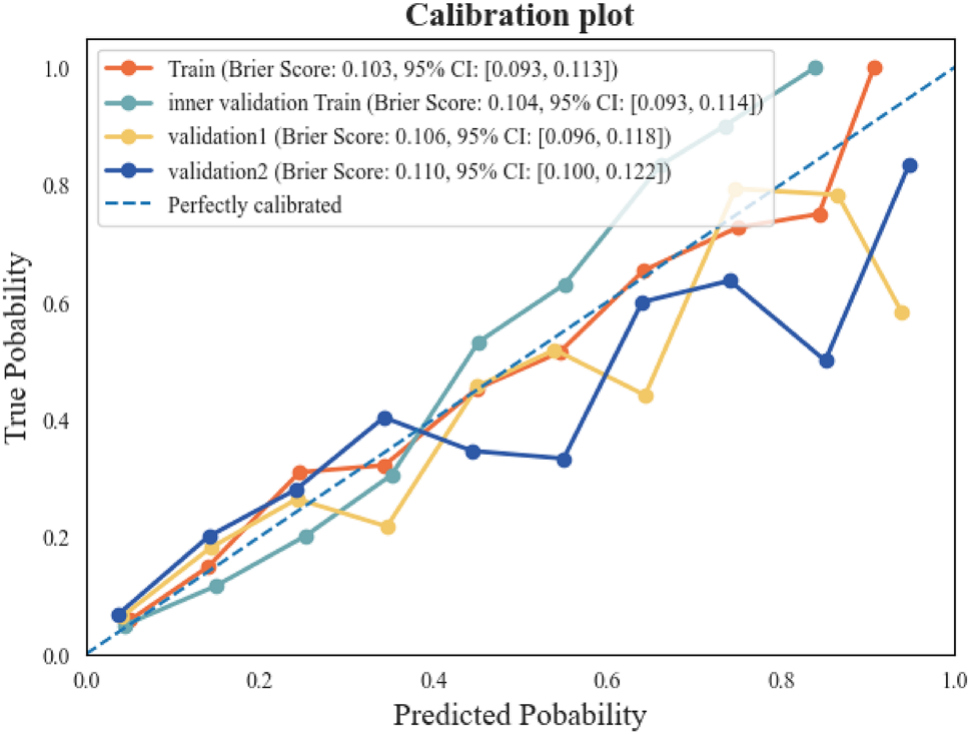
Calibration curves and calculated Brier scores. Red indicates training, green internal validation set, yellow validation 1, and blue validation 2. *CI* confidence interval.

To achieve a more comprehensive evaluation of the model’s performance, calibration curves and Brier scores were employed. The results are presented in Fig. 3 and Table 4. The Brier scores (with 95% CI) indicated good calibration, with values of 0.103 (0.093–0.113) for the training set, 0.106 (0.096–0.118) for validation set 1, and 0.110 (0.100–0.122) for validation set 2 (Table 4).

**Table 4:** Model evaluation of predicted AKI using 95% CI for AUC.

	Single- tier feature selection		Two-tier feature selection		
	Boruta	XGBoost	Train	Validation 1	Validation 2
AUC (95% CI)	0.83 (0.828–0.831)	0.85 (0.846–0.854)	**0.91 (0.906–0.915)**	0.83 (0.830–0.833)	0.85 (0.845–0.853)
Brier (95% )	0.121 (0.115–0.131)	0.211 (0.197–0.227)	**0.103 (0.093–0.113)**	0.106 (0.096–0.118)	0.110 (0.100–0.122)

*AUC* Area under the curve, *CI* confidence interval.

### Model interpretability

**Figure 4:**
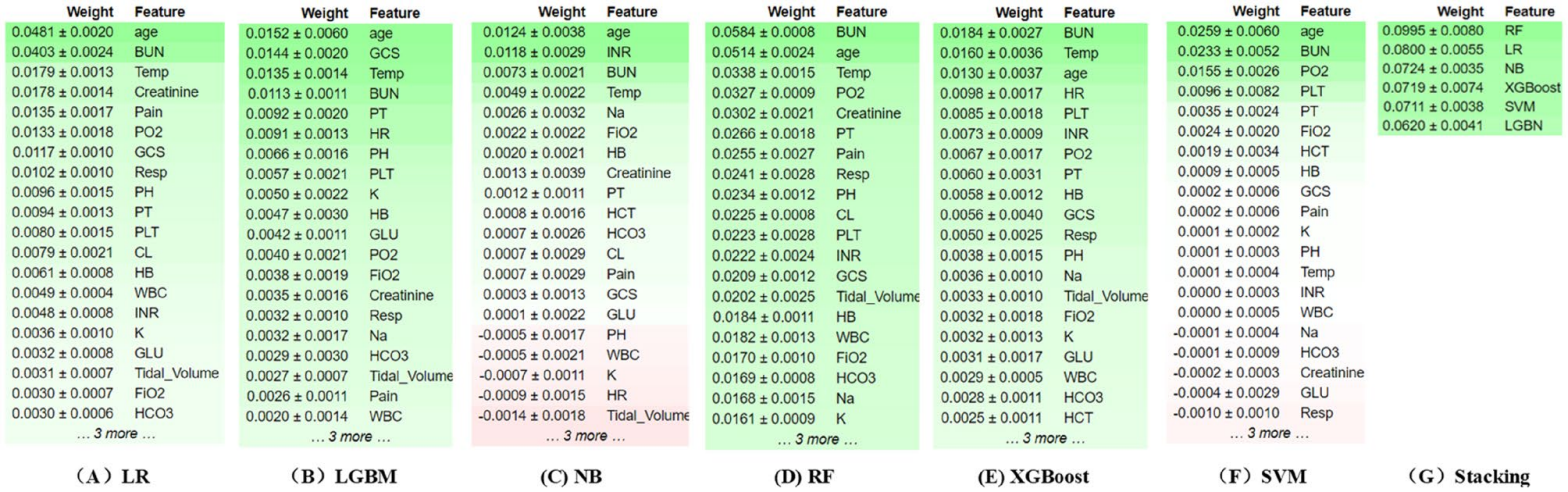
Feature importance analysis of stacking base models. (**A**) LR: Permutation Importance; (**B**) LGBM: Permutation Importance; (**C**) NB: Permutation Importance; (**D**) RF: Permutation Importance; (**E**) XGBoost: Permutation Importance; (**F**) SVM: Permutation Importance: The number next to each feature indicates the importance of that feature. A positive value indicates that the feature contributes positively to the model, while a negative value indicates that the feature contributes negatively to the model. The larger the number (positive or negative), the greater the influence of the feature on the model. Green indicates that the feature contributes positively to the model. Red indicates that the feature contributes negatively to the model. The shades of green and red represent the weights**.**

**Figure 5:**
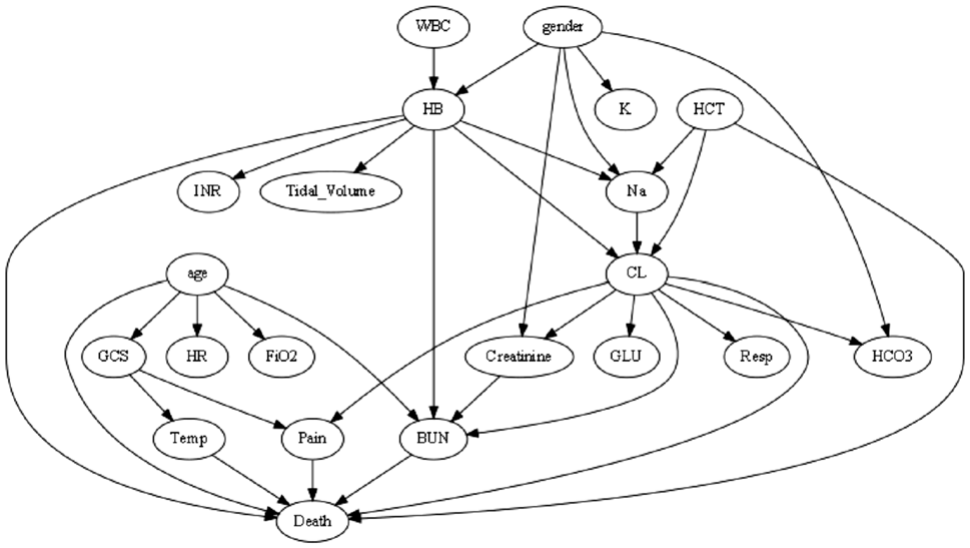
Model causal diagram.

**Figure 6:**
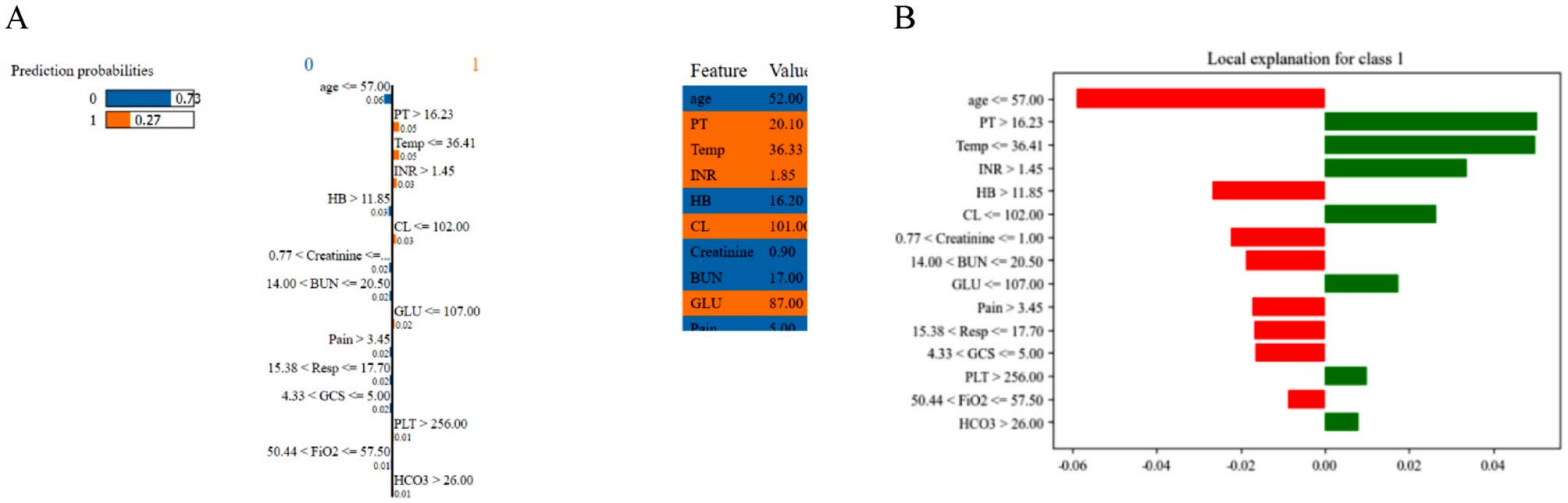
Interpretation of the predictions using the LIME algorithm with different random state values. (**A**) Predicted values for the model's disaggregated categories, feature coefficients (orange and blue indicate positive and negative relationships, respectively), and feature values in this sample are plotted against (**B**) local interpretations of features (red and green indicate positive and negative relationships, respectively).

The ensemble stacking model with two-tier feature selection utilizes two perspectives for model interpretation: individual and global. From an individual perspective, the interpretation module analyzes feature weights of the base model using the PI technique, as depicted in Fig. 4A–G. The top three important features in the base model are age, BUN, and temperature. During the analysis of the meta-model's feature weights, the predicted outputs of RFs' predicted outputs significantly influenced the final predictions. Table 5 displays the feature weights of the stacking model, which align with the findings of the base model analysis. From a global perspective, a causal diagram based on significant features is presented in Fig. 5. A causal relationship is observed between CL and HB as confounders of BUN and age and BUN as confounders of death, This relationship is represented as CL, HB → BUN → Death. However, determining the specific impact of each detection value is not feasible. Therefore, the influence of specific feature values is analyzed using LIME (Fig. 6). The LIME analysis reveals that the model's predictions vary under different combinations of feature values. For instance, the model is more likely to predict a lower risk of death when BUN ranges from 14.0 to 20.5 and age is ≤ 57 years, while predicting a higher risk of death when INR exceeds 1.5. This personalized analysis helps physicians understand the causal relationships between features and characteristics during model executions, thereby enhancing their comprehension of the models' decision-making processes.

**Table 5:** Overall model weights.

Feature	Weight
Age	0.02973 ± 0.0031
BUN	0.02650 ± 0.0018
Temp	0.01437 ± 0.0037
PO_2_	0.01203 ± 0.0020
PT	0.00931 ± 0.0033
PLT	0.00901 ± 0.0017
Creatinine	0.00877 ± 0.0025
INR	0.00768 ± 0.0018
PH	0.00717 ± 0.0029
Pain	0.00708 ± 0.0022
Resp	0.00692 ± 0.0013
HB	0.00631 ± 0.0019
CL	0.00518 ± 0.0016
WBC	0.00458 ± 0.0023
K	0.00455 ± 0.0022

In summary, the stacking ensemble model with two-tier feature selection integrates individual and global perspectives for model interpretation. The analysis of feature weights using the PI technique for both base and stacked models, along with causal diagrams and LIME analyses based on causal inference, enhances understanding of the model's predictive process and provides reliable references for medical decision-making.

## Discussion

In the building of predictive models for AKI, logistic regression with backward or forward selection is a common approach for selecting a subset of features for model construction^21^. More recent approaches, methods such as Lasso, Boruta^22^, and XGBoost^23^ have been employed for feature selection in AKI prediction.

However, Lasso methods are typically limited in their adaptability, often relying on linear models or assumptions. In nonlinear scenarios, Lasso methods may fail to accurately capture complex feature relationships, resulting in the selection of insufficient features for effective data interpretation. Logistic regression, which relies on a linear combination of individual features for classification, may not adequately capture feature interactions, potentially impacting prediction accuracy. While Boruta^6^, a feature selection method based on tree models, excels at uncovering complex feature relationships and handling highly correlated features. Nonetheless, it solely focuses on the relationship between features and targets, disregarding the importance of features and models. XGBoost^24^, a gradient-boosting tree model, excels at capturing complex relationships among features, particularly in nonlinear scenarios. Its feature selection process focuses on the correlation between features and the model.

Several studies have highlighted the importance of feature selection in improving model performance for AKI prediction. Zhou et al.^25^ demonstrated significant improvements in model predictions by incorporating deep features alongside those extracted using convolutional neural networks (CNNs). Similarly, Zhu et al.^19^ observed a substantial enhancement in prediction accuracy following a 71% reduction in feature set size. Based on these findings, we propose that the model prediction performance can be improved by selecting intersecting features and reducing redundant features. Therefore, in our experiments, we first conducted feature selection using the Boruta algorithm to filter out features that correlate with the target value. Subsequently, in the second tier of feature selection, we employed XGBoost to filter out features that correlate with the model. Experimental results demonstrate the superiority of the two-tier feature selection approach over the single-tier approach. The stacking ensemble model exhibited superior predictive performance compared to the baseline model. Notably, the stacking ensemble model with two-tier feature selection achieved the highest predictive performance. Yue et al.^26^ applied the Boruta algorithm to screen 34 variables and built a random forest model for predicting mortality risk in acute kidney injury patients, achieving an AUROC of 0.82. Yang et al.^27^ employed the Boruta algorithm to select 36 variables and utilized XGBoost for modeling mortality risk prediction in sepsis-associated acute kidney injury patients, achieving an AUROC of 0.85. In comparison to previous studies, our proposed model achieved an AUROC of 0.91, indicating improved model performance. These results affirm the efficacy of our proposed approach. Furthermore, by employing model interpretable techniques and causal inference, we conducted a causal analysis of factors influencing model predictions. Our findings revealed significant associations between various laboratory tests and the prediction of mortality risk in AKI patients, consistent with previous studies by Son^28^, Zhang^29^, and others, further validating the reliability of our model. Taken together with the experimental results, the two-tier feature selection proposed in this study can better predict the risk of death of AKI patients in the ICU, and it can better capture the complexity and diversity of AKI risk by reducing the confounding variables in the model inputs. By combining the predictive power of multiple models, it can provide a more reliable auxiliary diagnosis for clinical decision-making.

However, it is worth noting that a common challenge we faced was that this study was not prospective but retrospective. We chose to use the MIMIC-III dataset, but it is important to note that this dataset does not adequately represent the entire population and the diversity of different clinical practices. This somewhat limits our ability to analyze the problem in depth and make accurate predictions, as well as our ability to generalize the model to real-world applications. It is worth noting that due to the large number of missing urine output indicators in the dataset, we chose to temporarily omit this indicator from our study after referring to the relevant literature^18,30–33^. However, recent studies^34^ suggest that urine output plays a crucial role in the disease progression of AKI. Therefore, we will fully consider urine output as an important indicator in future studies to improve the ability to predict more accurately the risk of death in patients.

## Methods

**Figure 7:**
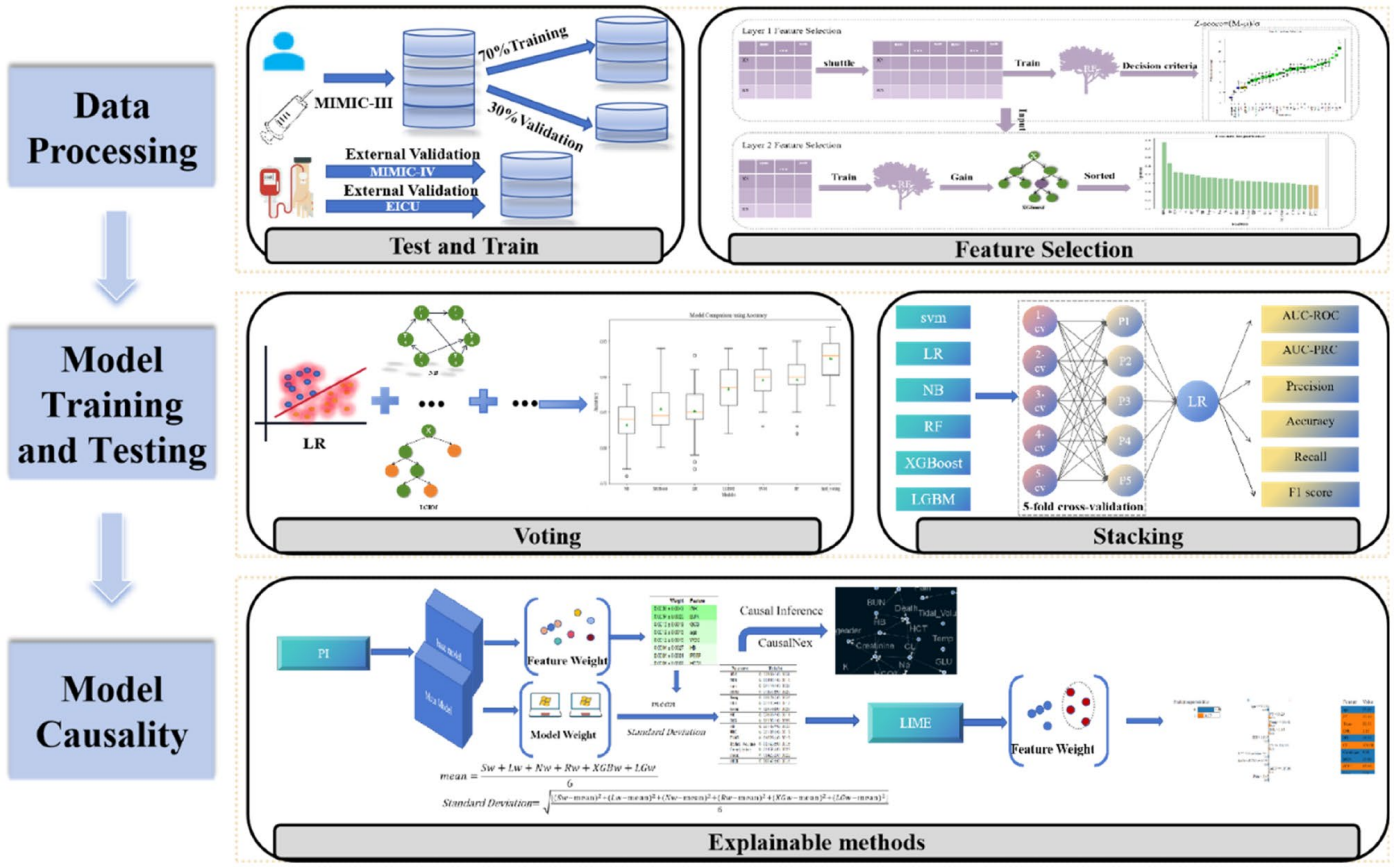
A conceptual model for predicting outcomes in AKI patients within the ICU using a limited set of features. The initial conceptual model is designed for ongoing prediction of AKI-related hospitalization outcomes. Firstly, we gather data on the patient's laboratory tests, surgeries, and medication usage. Secondly, relevant features are identified for prediction through feature selection. Thirdly, we introduce a stacking ensemble model, employing fivefold cross-validation to assess patient outcomes. Lastly, the model undergoes analysis using various interpretable methods.

The conceptual framework for our developing two-tier feature selection prediction model is presented in Fig. 7.

### Study population

Data for this study were retrieved from three distinct critical care databases: MIMIC-III^35^, MIMIC-IV^36^, and eICU-CRD^37^. The prediction models were developed using the publicly accessible MIMIC-III databases. The data were divided into two sets: 30% of the data were reserved for internal validation, and the remaining 70% were used for model construction. The predictive performance of these models was validated using an entirely independent dataset, the MIMIC-IV and eICU-CRD datasets. MIMIC-III includes critical care data from 46,520 ICU patients admitted to Beth Israel Deaconess Medical Center in Boston between June 1, 2001, and October 31, 2012. This dataset encompasses 26 tables encompassing demographics, admission records, discharge summaries, ICD-9 diagnostic records, vital signs, laboratory measurements, and medication usage. In contrast, MIMIC-IV includes data from over 190,000 patients, 450,000 hospitalizations, and more than 1000 hospital admissions to Beth Israel Deaconess Medical Center (BIDMC) and Massachusetts Institute of Technology (MIT) between 2008 and 2019, totaling 1,000,000 admissions. It offers a broader array of information, covering demographics, laboratory tests, medication usage, vital signs, surgical procedures, and disease diagnoses. Although MIMIC-III and MIMIC-IV may share medical information and data types, their data collection, processing, and dissemination methodologies differ. The MIMIC-IV dataset is broader in scope, spanning more hospitals and patients and covering a longer timeframe. The eICU-CRD Collaborative Research Database (eICU-CRD) is a large public database created by MIT in collaboration with the Laboratory for Computational Physiology (LCP). The database is a completely independent dataset that brings together data from many hospitals within the United States, expanding the scope of the study by providing data from multiple centers. The database covers routine data on more than 200,000 patients admitted to intensive care units in 2014 and 2015 and includes a wealth of high-quality clinical information such as physiological parameters, laboratory results, medication records, and diagnostic information. The data are presented in both structured and unstructured forms and are automatically collected from monitoring equipment, electronic medical records, and other healthcare information systems.

For each patient sample, the following information was collected: (1) Demographic characteristics: including gender, age in years, and survival status; (2) Vital signs: including heart rate (HR, beats/min), respiratory rate (Resp, beats/min), body temperature (Temp, degrees Celsius), and pain (pain, not applicable); (3) Laboratory parameters: including blood urea nitrogen (BUN, mg/dL), creatinine (Creatinine, mg/dL), glucose (GLU, mg/dL), bicarbonate (HCO_3_, mmol/L), international normalized ratio (INR), potassium (K), potassium (K, mmol/L), sodium (Na, mmol/L), partial pressure of carbon dioxide (PCO_2_, mmHg), prothrombin time (PT, s), white blood cell count (PCR, mmol/L s), white blood cell count (WBC, in 10^3^/μL), chloride (CL, in mmol/L), Glasgow Coma Scale (GCS), hematocrit (HCT, %), hemoglobin ( HB, g/dL), acid–base balance index (PH,) platelet count (PL, in mmol/L), platelet count (PLT, in 10^3^/μL), oxygen pressure (PO_2_, in mmHg), peripheral oxygen saturation (SpO_2_, in %), and fraction of inspired oxygen (FiO_2_, in %). Blood samples were taken before and after dialysis, following an 8-h fast for routine biochemical testing.

### Determination of outcome variables: mortality and AKI

Mortality, defined as the death rate among patients with AKI during their ICU hospitalization, was determined through specific criteria. Firstly, AKI diagnosis followed the Kidney Disease Improving Global Prognosis (KDIGO)^1^ guidelines, considering serum creatinine concentration (Scar) and urine output (UO) levels. According to literature studies^30–33,38^, serum creatinine concentration (Scar) was used as the main target of study in this experiment. AKI was defined as: a 1.5-fold increase in serum creatinine concentration within the prior 7 days; a rise of ≥ 0.3 mg/dL within 48 h; or a sustained urine output of < 0.5 mL/kg/h for ≥ 6 h. In cases where baseline serum creatinine was unavailable pre-admission, the first serum creatinine at admission served as the baseline. Patients with AKI in the ICU were identified via departmental codes. Subsequently, ICU duration was computed based on admission and discharge times, and data from 24 h preceding admission were extracted^26,39,40^. Data from the initial ICU admission were used for patients with multiple admissions; the average value was calculated for repeated examinations within 24 hours^41^.

### Inclusion and exclusion criteria

**Figure 8:**
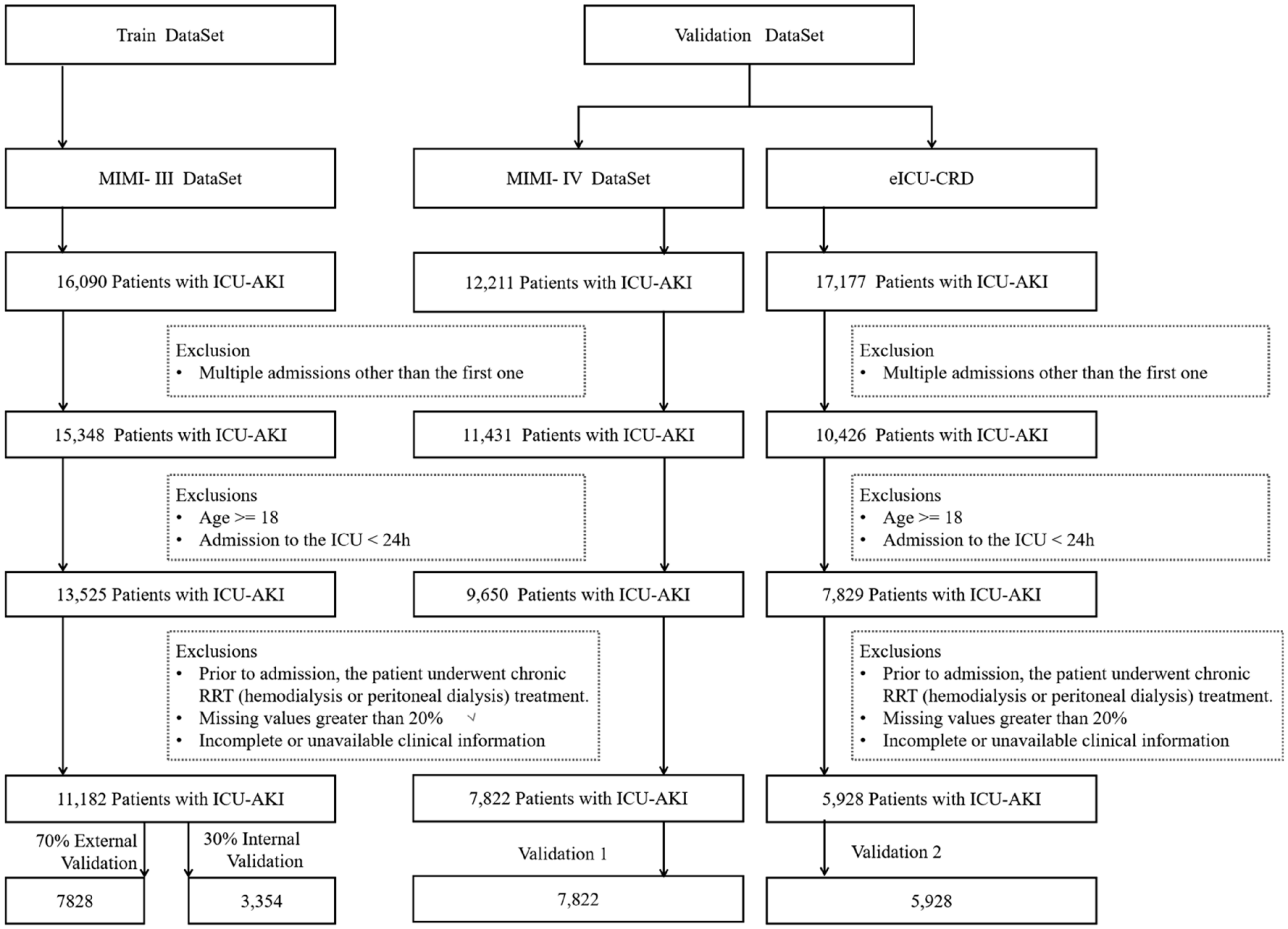
Flow chart of the study population selection. ICU-AKI: patients with acute kidney injury (AKI) treated in the Intensive Care Unit (ICU).

To ensure data safety and emphasize the effectiveness of the model in early prediction, we focused on developing a predictive model using medical data from 24 h prior to a patient's admission to the hospital to screen patients diagnosed with AKI. The final dataset for the experiment was selected from this data (Fig. 8). During the data selection process, we excluded patients who met the following criteria: (1) age < 18 years old; (2) patients who were admitted to the intensive care unit for > 24 h; (3) patients who had already received chronic renal replacement therapy prior to admission; and (4) data with < 20% of missing values or a lack of outcome information. These exclusion criteria were designed to ensure the quality and accuracy of the experimental data for better exploring the relationship between early patient status and AKI.

### Data processing

In this study, datasets with missing values exceeding 20% were excluded, and outliers were identified using box-and-whisker plots and subsequently removed. To handle missing values, multiple imputations were performed utilizing the RF algorithm, known for its effectiveness in imputing missing data^42^ RF offers several advantages, including the ability to handle mixed types of missing data, adaptability to interactions and nonlinearities, and scalability to large datasets^43^, while preserving the distribution of data post-imputation. Additionally, the data underwent Min–Max normalization, transforming it into a specific range of intervals to ensure uniform scaling of each feature. This normalization process ensured uniform scaling for each feature, maintaining a relative weight balance between features. By addressing issues of model bias towards certain features due to differing scales, the normalization process improved the performance and interpretability of the machine learning model, ensuring consistent contribution weights of individual features to the model.

### Statistical analyses

Descriptive statistics were utilized to assess the distribution and inherent patterns of numerical characteristics within the dataset. Measures such as mean, median, mode, range, variance, and standard deviation were examined as appropriate. Pearson's correlation coefficient was employed to analyze the degree of linear correlation between variables. Descriptive statistics for continuous variables included either mean ± standard deviation or median (interquartile range), while frequencies were utilized for categorical variables. The normal distribution of each variable was evaluated using the Kolmogorov–Smirnov test. Student's t-test compared continuous variables, and Fisher's exact method was used for correlational analysis between variables. Statistical analysis was performed using R version 4.3.1 for Windows.

### Feature selection

This study employs a two-tier feature selection approach to improve both the performance and interpretability of the prediction model. The Boruta algorithm was utilized in the initial tier, and the XGBoost algorithm was employed in the subsequent tier. Boruta^6,44^ is a RF-based feature selection method that evaluates feature importance through modelling the distribution of random and original features. In the first tier, Boruta is applied to filter out features with significant predictive power for the target variable from the initial set. XGBoost^23^, an efficient gradient boosting tree algorithm, serves as the meta-model in the second tier, known for its excellent predictive performance and automatic feature screening. Feature selection within the XGBoost model further refines the initially selected features, resulting in a final subset with enhanced predictive power and stability.

### Model construction

In this study, we have strategically employed the SEM (Stacking Ensemble Method) to build our model, with the goal of further enhancing overall model performance by adeptly integrating outputs from multiple base learners (single classifiers) as inputs to the meta-learner. Extensive prior research has demonstrated the substantial superiority of the SEM in performance compared to independent classifiers^45^. To further optimize model performance, this study has employed the voting ensemble method in the preliminary stage. This method selectively crafts the base model for the SEM based on data characteristics and the principle of model diversity. Ultimately, Logistic Regression (LR), Support Vector Machine (SVM), Naive Bayes (NB), Light Gradient Boosting Machine (LGBM), EXtreme Gradient Boosting (XGBoost), and Random Forest (RF) were identified as the base models for stacking ensemble, with LR being specifically chosen as the metamodel. This decision was made considering that the variables outputted by the base models represent linear data and align with the pursuit of model interpretability. The objective of this selection is to strike a balance between the diversity of the base models and the performance of the overall model, thereby providing a more comprehensive and reliable analytical foundation for this study.

### Evaluation metrics

To assess the performance of the used models comprehensively and thoroughly, we utilize a diverse set of performance metrics, encompassing the area under the Receiver Operating Characteristic (AUROC), 95% Confidence Interval (CI), Precision-Recall Curve (AUC-PRC), Precision, Accuracy, Recall, F1 score, Calibration curves, and Brier scores. This comprehensive metric framework is designed to provide a more holistic understanding of the model's performance across various dimensions. Specifically, the evaluation is conducted using the following formulas:

True Positive (TP), True Negative (TN), False Positive (FP), and False Negative (FN).

1 $$AUC-ROC={\int }_{0}^{1}TPd FP$$

2 $$AUC-PRC={\int }_{0}^{1}FPd Recall$$

3 $$Recall=\frac{TP}{TP+FN}$$

4 $$Precision=\frac{TP}{TP+FP}$$

5 $$Accuarcy=\frac{TP+TN}{TP+TN+FP+FN}$$

6 $$F1 score=\frac{2\times Precision\times Recall}{Precision+Recall}$$

7 $$Brier Score=\frac{1}{N}\sum_{i=1}^{N}{({f}_{i}-{o}_{i})}^{2}$$

N is the total number of samples, ${f}_{i}$ is the predicted probability of the ith predicted sample, and ${o}_{i}$ is the actual outcome of the ith sample (usually 0 or 1).

### Model interpretability

The SEM combines multiple base models to generate predictions. Thus, when interpreting the model, the feature weights of each base model undergo an initial assessment using the Permutation Importance (PI) technique. Subsequently, mathematical computation determines the feature weights of each model, which are then utilized as the feature weights of the stacked models. Features with higher weights are selected based on importance ranking, and a causal diagram is constructed using a causal inference framework^46^. In this framework, confounders are defined as variables directly influencing both the predicted outcome and the predictor. These confounders are pivotal factors contributing to AKI mortality rates^47^. Finally, Local Interpretable Model-Agnostic Explanations (LIME) is employed to analyze how specific values of different characteristics impact the model's predicted outcomes across various categories. This elucidation of clinical parameters leading to high patient mortality facilitates targeted interventions for potentially critical illnesses during clinical practice.

### Supplementary Information

Supplementary Information.

## Data Availability

The datasets generated during the current study are available in the GitHub (https://github.com/mengqings/Data_aki_all/tree/master and https://github.com/mengqings/eICU_Data_extract/tree/master) repository.
