# Supplementary material for: A two-tier feature selection method for predicting mortality risk in ICU patients with acute kidney injury
Source: Sci Rep. 2024 Jul 22;14:16794. doi: 10.1038/s41598-024-63793-3 (PMC11263702; doi:10.1038/s41598-024-63793-3)
Supplement: Supplementary file 1 — Supplementary Information. [file 41598_2024_63793_MOESM1_ESM.docx]

| A  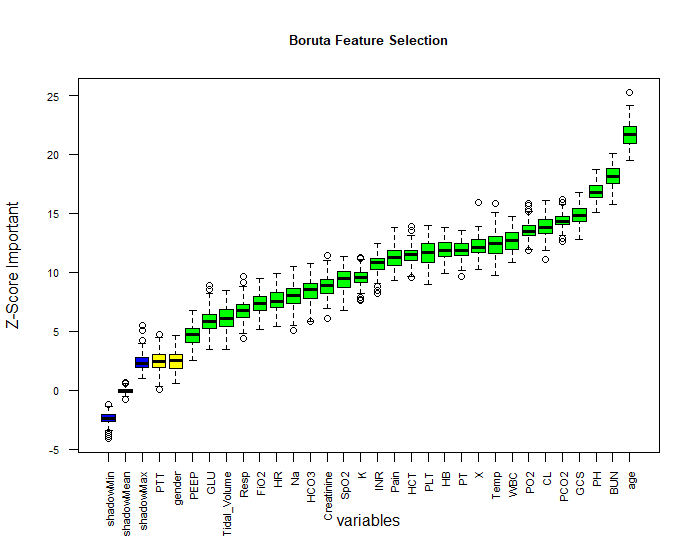 | | B  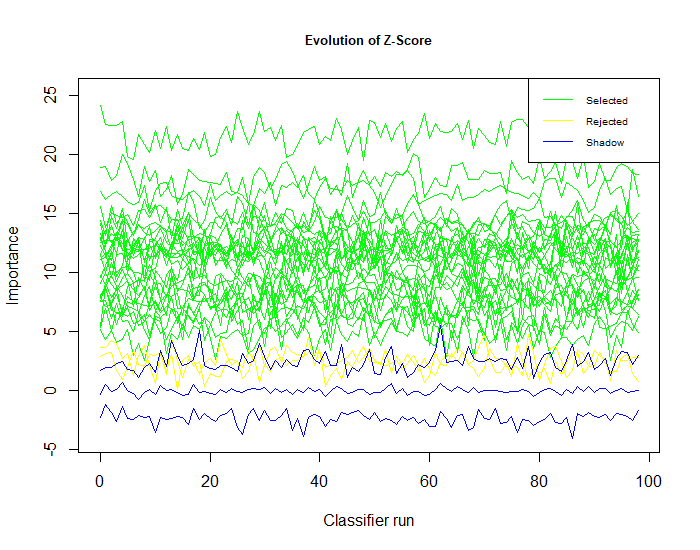 | |
| --- | --- | --- | --- |
| **FIG 1** \| (A) feature selection based on Boruta's algorithm. (B) Different features' performance in the classifier. Green color indicates selected features, yellow color indicates discarded features, and blue color indicates the maximum, minimum, and average of z-score scores for shaded features. | | | |
| A  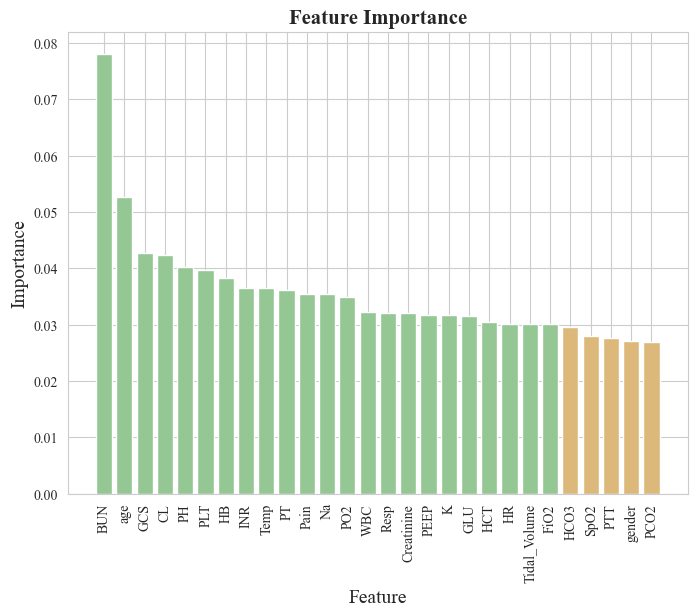 | | B  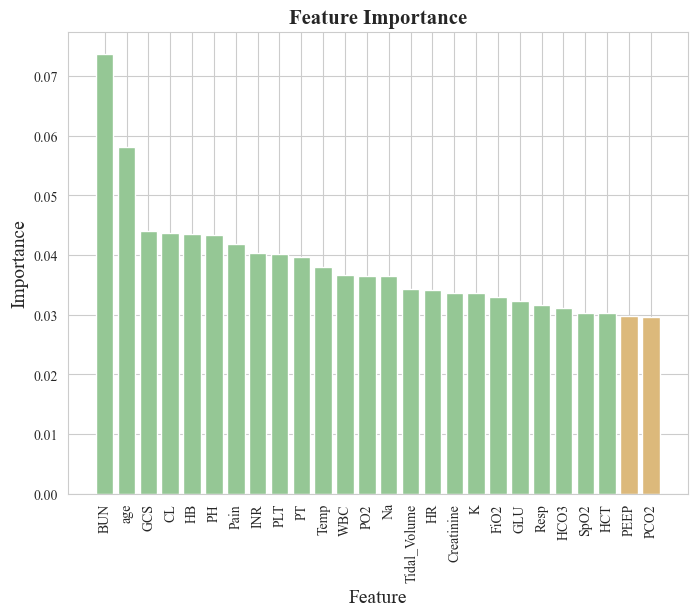 | |
| **FIG 2** \| (A) XGBoost feature selection. The horizontal axis is the name of each feature variable, and the vertical axis is the importance of each variable. (B) After the first layer of feature selection, the second layer of feature selection results. Green indicates selected features; other colors indicate redundant features. | | | |
| **Internal Validation** | | | |
| A  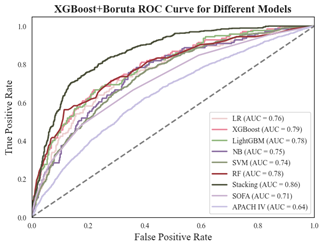 | B  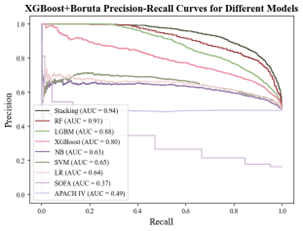 | | C  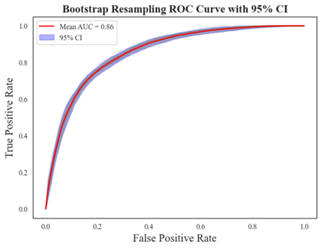 |
| **FIG 3 \| Internal validation of model performance.** | | | |

| A  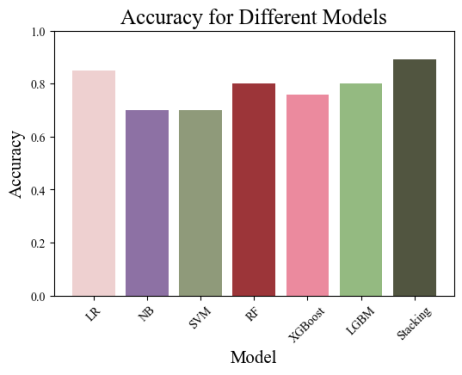 | B  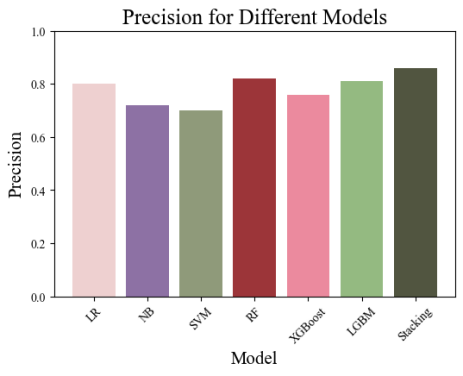 | C  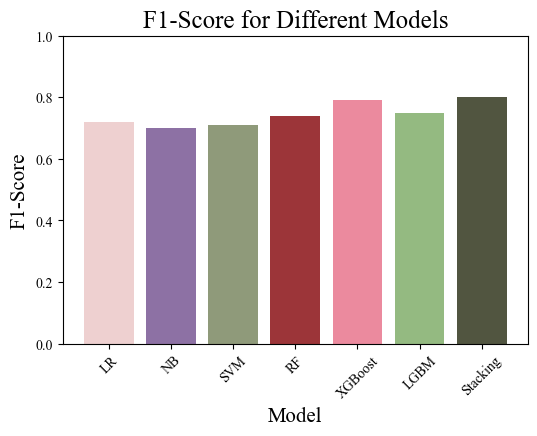 |
| --- | --- | --- |
| **FIG 4** \| Internal Validation set precision, accuracy, and f1 scores for the best performing model . (A)：Accuracy; (B): Precision； (C) F1-Score. | | |

| **Characteristics** | **Train DataSet**  **7828** | | **Internal Validation**  **3354** | | **Validation**  **7822** | |
| --- | --- | --- | --- | --- | --- | --- |
|  | **Survival**  **5555** | **Death**  **2273** | **Survival**  **2514** | **Death**  **840** | **Survival**  **6705** | **Death**  **1117** |
| Age (%) |  |  |  |  |  |  |
| 18<age<30 | 198 (3.56) | 27 (1.18) | 69 (2.74) | 4 (0.47) | 267 (3.98) | 19 (1.70) |
| 30<age< 60 | 1880 (33.84) | 517 (22.74) | 798 (31.74) | 193 (22.98) | 3075 (48.86) | 443 (39.65) |
| >60 | 3477 (62.59) | 1729 (76.07) | 1647 (65.51) | 643 (76.55) | 3363 (50.15) | 655 (58.63) |
| Gender (%) |  |  |  |  |  |  |
| Female | 2411 (43.4) | 1038 (45.7) | 1079 (42.9) | 359 (42.7) | 2827 (42.2) | 505 (45.2) |
| Male | 3144 (56.6) | 1235 (54.3) | 1435 (57.1) | 481 (57.3) | 3878 (57.8) | 612 (54.8) |
| BUN (mmol/L) | 24.09 (16.04) | 22.95 (14.89) | 33.70 (19.17) | 33.63 (18.70) | 24.74 (16.48) | 31.84 (18.19) |
| CL (mmol/L) | 105.54 (5.32) | 105.78 (5.19) | 104.96 (6.37) | 105.01 (6.17) | 100.10 (3.71) | 100.38 (3.87) |
| Creatinine(mg/dL) | 1.32 (1.07) | 1.27 (1.03) | 1.65 (1.16) | 1.78 (1.30) | 1.41 (1.33) | 1.54 (1.21) |
| FiO2 (%) | 60.56 (22.93) | 60.12 (23.09) | 60.56 (22.93) | 60.12 (23.09) | 60.56 (22.93) | 60.12 (23.09) |
| GCS | 4.07 (1.05) | 4.03 (1.05) | 3.34 (1.46) | 3.42 (1.43) | 14.79 (1.04) | 14.53 (1.62) |
| GLU (mmol/L) | 133.25(40.32) | 132.41 (37.54) | 144.30 (46.88) | 142.70 (47.24) | 100.10 (3.71) | 100.38 (3.87) |
| HCO3 (mmol/L) | 18.05 (8.45) | 18.29 (8.30) | 18.05 (8.45) | 18.29 (8.30) | 18.05 (8.45) | 18.29 (8.30) |
| HCT (%) | 31.92 (5.35) | 31.96 (5.31) | 31.90 (5.96) | 31.69 (5.72) | 34.15 (6.22) | 32.92 (6.56) |
| HR (bpm) | 84.80 (15.05) | 84.27 (14.67) | 87.70 (17.14) | 88.44 (17.17) | 85.10 (15.23) | 85.99 (16.10) |
| HB (g/L) | 10.79 (1.81) | 10.85 (1.79) | 10.64 (2.03) | 10.51 (1.85) | 11.51 (3.21) | 11.39 (3.30) |
| INR | 1.34 (0.53) | 1.31 (0.46) | 1.55 (0.69) | 1.56 (0.71) | 1.32 (0.62) | 1.42 (0.84) |
| K (mmol/L) | 4.19 (0.99) | 4.24 (3.28) | 4.27 (0.68) | 4.26 (0.65) | 5.27 (2.03) | 5.31 (2.01) |
| Na (mmol/L) | 138.34 (3.68) | 138.25 (3.67) | 138.31 (4.87) | 138.35 (4.79) | 136.50 (14.18) | 136.44 (14.14) |
| PCO2 (mmHg) | 47.80 (10.36) | 48.34 (11.46) | 46.22 (12.56) | 45.77 (11.73) | 47.20 (10.30) | 47.97 (11.29) |
| PEEP (cmH2O) | 5.90 (1.15) | 5.87 (1.17) | 6.39 (1.60) | 6.41 (1.53) | 5.95 (1.23) | 6.20 (1.30) |
| PH | 6.71 (0.77) | 6.71 (0.78) | 6.74 (0.75) | 6.75 (0.72) | 6.76 (0.75) | 6.61 (0.77) |
| PLT (10^9^/L]) | 207.53(88.25) | 207.73 (84.94) | 197.73 (103.13) | 194.53 (105.55) | 248.35 (138.17) | 224.98 (114.87) |
| PO2 (mmHg) | 140.56(61.22) | 144.63 (62.31) | 129.93 (55.62) | 126.46 (55.62) | 143.14 (62.51) | 122.65 (52.23) |
| PT (s) | 15.62 (45.17) | 15.27 (24.16) | 17.43 (8.90) | 17.63 (9.67) | 14.56 (6.58) | 15.58 (8.93) |
| PTT (s) | 35.70 (12.22) | 35.41 (12.28) | 39.43 (14.93) | 40.22 (14.91) | 36.17 (12.57) | 38.62 (13.73) |
| Pain | 2.11 (2.13) | 2.12 (2.18) | 1.19 (1.81) | 1.27 (1.84) | 2.13 (2.16) | 1.56 (1.92) |
| Resp (bpm) | 18.00 (3.97) | 18.04 (3.96) | 19.40 (4.44) | 19.53 (4.60) | 11.53 (9.55) | 13.16 (10.42) |
| SpO2 (%) | 96.96 (5.00) | 97.13 (9.00) | 96.26 (3.36) | 96.29 (3.86) | 87.43 (15.43) | 87.16 (15.16) |
| Temp (°C) | 36.77 (0.54) | 36.76 (0.53) | 36.68 (0.71) | 36.63 (0.70) | 37.38 (1.14) | 37.28 (1.33) |
| Tidal Volume (mL) | 491.96(75.82) | 491.83 (74.88) | 480.15 (79.71) | 484.68 (77.49) | 475.52 (82.10) | 487.58 (75.49) |
| WBC (cells/μL) | 11.45 (5.09) | 11.53 (5.04) | 12.70 (6.82) | 12.53 (6.81) | 11.63 (5.43) | 11.45 (5.09) |
| **Table 1 \| The baseline characteristics of AKI patients are analyzed.** Statistical analysis of mortality and survival data within the training, internal validation, and external validation sets is conducted. Age and gender are depicted as frequencies and proportions within various categories, whereas other indicators are expressed using their mean values and standard deviations. | | | | | | |

|  | **XGBoost** | | | | |  | **Boruta** | | | | |
| --- | --- | --- | --- | --- | --- | --- | --- | --- | --- | --- | --- |
| **Models** | **AUC** | **Accuracy** | **Precision** | **Recall** | **F1 score** |  | **AUC** | **Accuracy** | **Precision** | **Recall** | **F1 score** |
| SVM | 0.70 | 0.70 | 0.69 | 0.70 | 0.70 |  | 0.74 | 0.72 | 0.70 | 0.78 | 0.76 |
| NB | 0.68 | 0.70 | 0.72 | 0.72 | 0.70 |  | 0.74 | 0.73 | 0.82 | 0.78 | 0.72 |
| LR | 0.69 | 0.80 | 0.82 | 0.83 | 0.81 |  | 0.78 | 0.85 | 0.83 | 0.85 | 0.82 |
| RF | 0.78 | 0.82 | 0.82 | 0.79 | 0.80 |  | 0.80 | 0.86 | 0.84 | 0.86 | 0.82 |
| XGBoost | 0.77 | 0.83 | 0.82 | 0.83 | 0.82 |  | 0.81 | 0.86 | 0.84 | 0.84 | 0.83 |
| LGBM | 0.77 | 0.84 | 0.80 | 0.80 | 0.80 |  | 0.80 | 0.86 | 0.84 | 0.84 | 0.84 |
| **Stacking** | **0.85** | **0.85** | **0.84** | **0.83** | **0.81** |  | **0.83** | **0.87** | **0.86** | **0.82** | **0.85** |

**Table 2|** **Evaluation of models for predicting AKI with single-tier feature selection**. AUC: area under the curve.

|  | **Internal Validation** | | | | |
| --- | --- | --- | --- | --- | --- |
| **Models** | **AUC** | **Accuracy** | **Precision** | **Recall** | **F1 score** |
| SVM | 0.74 | 0.70 | 0.70 | 0.69 | 0.68 |
| NB | 0.75 | 0.70 | 0.72 | 0.71 | 0.70 |
| LR | 0.76 | 0.85 | 0.80 | 0.85 | 0.82 |
| RF | 078 | 0.80 | 0.82 | 0.82 | 0.78 |
| XGBoost | 0.79 | 0.76 | 0.76 | 0.75 | 0.76 |
| LGBM | 0.78 | 0.80 | 0.81 | 0.81 | 0.79 |
| Stacking | 0.86 | 0.89 | 0.86 | 0.81 | 0.82 |

**Table 3|** **Evaluation of models for predicting AKI with internal validation**
